# Supplementary figures and images for: “Integrative learning” promotes learning but not memory in older rats
Source: PeerJ. 2023 Mar 29;11:e15101. doi: 10.7717/peerj.15101 (PMC10066688; doi:10.7717/peerj.15101)

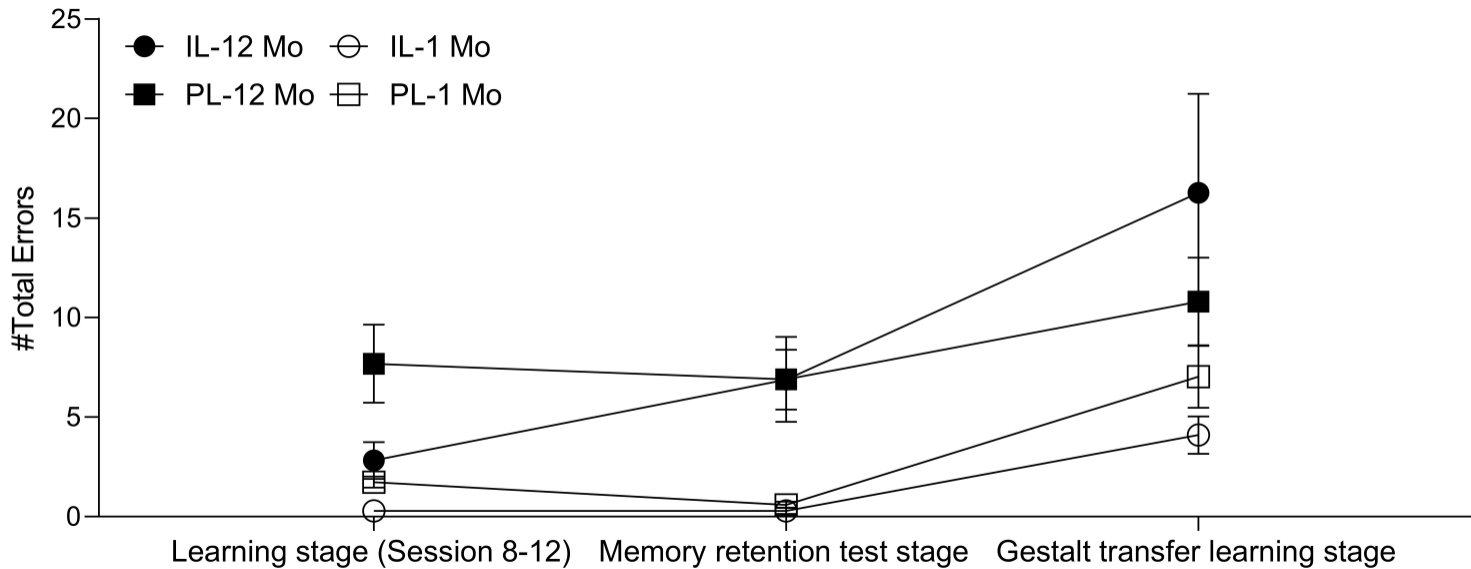

Supplement: Supplemental Information 1 — Each data point represents the average of total errors made by a single rat in each group during Sub-stage Three (Sessions 8–12) of the learning stage, the memory retention test stage and the Gestalt transfer learning stage. Error bar represents standard error. [file peerj-11-15101-s001.pdf]

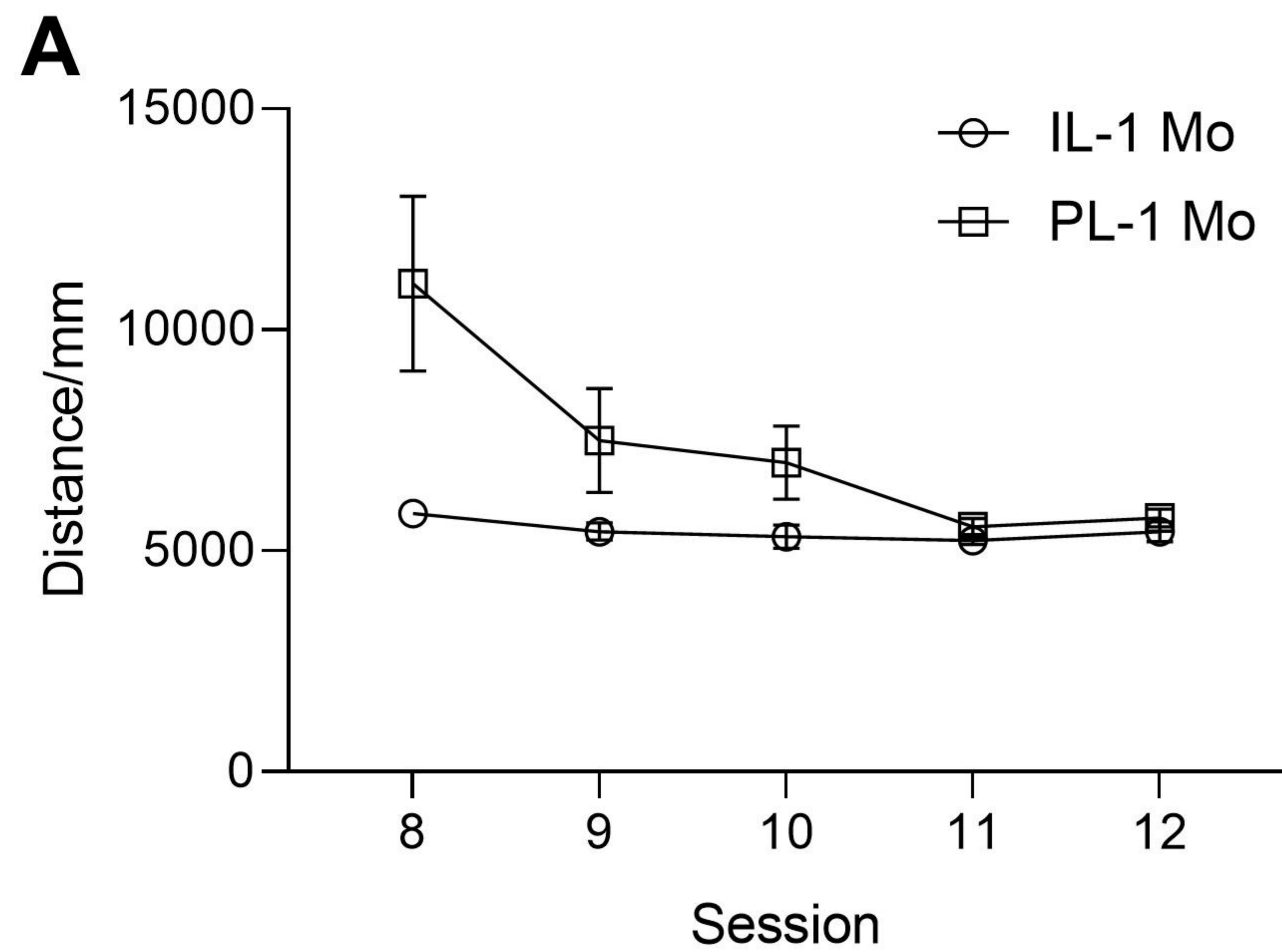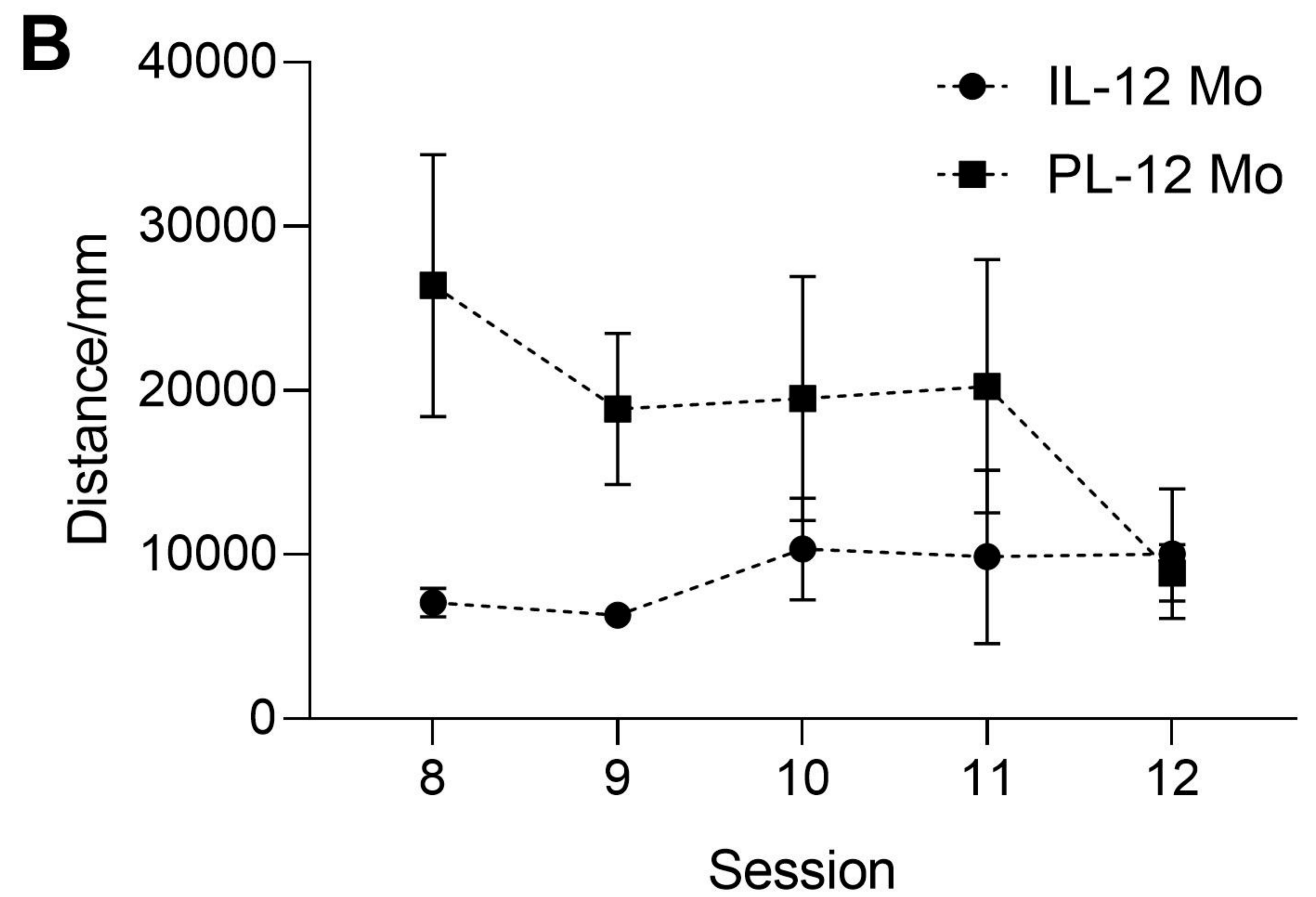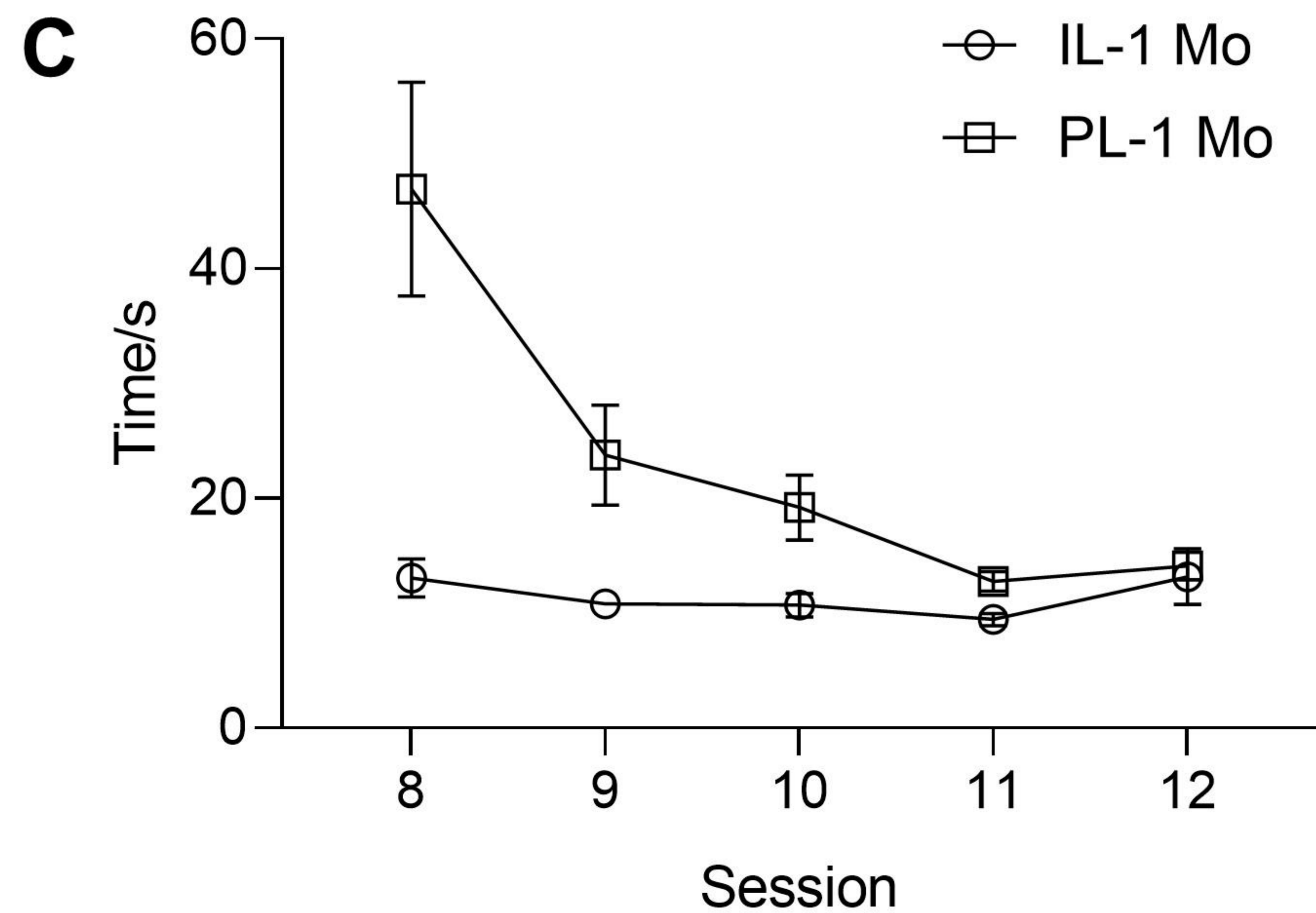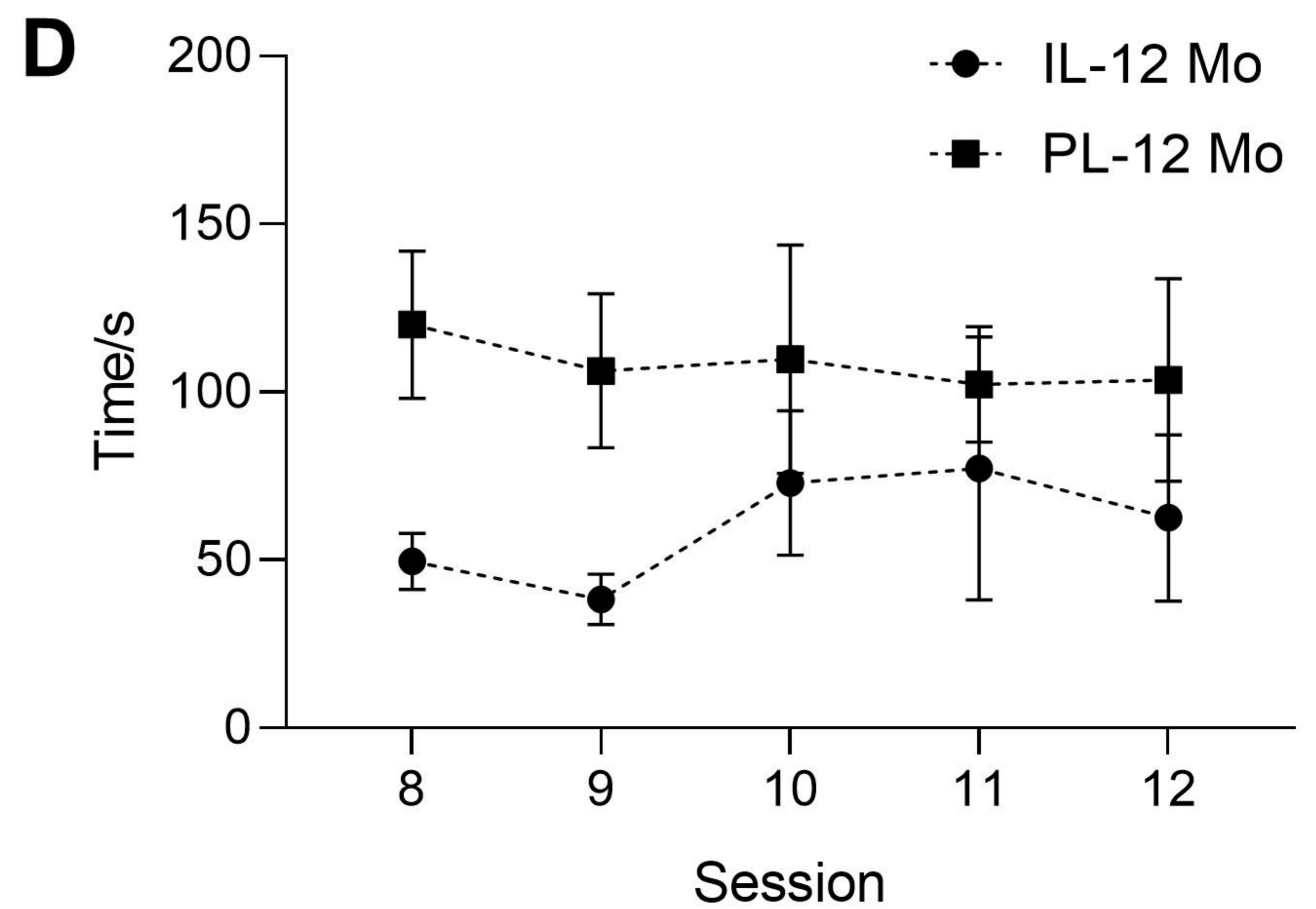

Supplement: Supplemental Information 2 — Upper panels (A, B) show total travel distince by different groups and lower panels (C, D) show total travel time during Sub-stage Three (Sessions 8–12) of the learning stage. Each data point represents the group average and the error bar represents SEM. [file peerj-11-15101-s002.pdf]
