# Supplementary material for: “Integrative learning” promotes learning but not memory in older rats
Source: PeerJ. 2023 Mar 29;11:e15101. doi: 10.7717/peerj.15101 (PMC10066688; doi:10.7717/peerj.15101)
Supplement: Supplemental Information 3 [file peerj-11-15101-s003.docx]

| Table S1 Simple-effect comparison on the total errors made between learning modes controlling for age and session (Learning Stage) | | | | | |
| --- | --- | --- | --- | --- | --- |
|  | *SS* | *DF* | *MS* | *F* | *p* |
| Learning mode within 1 Mo by mwithin Session 1 | 649.8 | 1 | 649.8 | 16.14 | .000*** |
| Learning mode within 12 Mo by mwithin Session 1 | 1150.75 | 1 | 1150.75 | 28.59 | .000*** |
| Learning mode within 1 Mo by mwithin Session 2 | 120.05 | 1 | 120.05 | 5.37 | .027* |
| Learning mode within 12 Mo by mwithin Session 2 | 133.2 | 1 | 133.2 | 5.96 | .021* |
| Learning mode within 1 Mo by mwithin Session 3 | 26.45 | 1 | 26.45 | 2.67 | .112 |
| Learning mode within 12 Mo by mwithin Session3 | 33.6 | 1 | 33.6 | 3.40 | .075 |
| Learning mode within 1 Mo by mwithin Session 4 | 68.45 | 1 | 68.45 | 5.50 | .026* |
| Learning mode within 12 Mo by mwithin Session 4 | 37.47 | 1 | 37.47 | 3.01 | .093 |
| Learning mode within 1 Mo by mwithin Session 5 | 57.80 | 1 | 57.80 | 1.89 | .179 |
| Learning mode within 12 Mo by mwithin Session 5 | 9.80 | 1 | 9.80 | .32 | .575 |
| Learning mode within 1 Mo by mwithin Session 6 | 1.25 | 1 | 1.25 | .09 | .766 |
| Learning mode within 12 Mo by mwithin Session 6 | 44.66 | 1 | 44.66 | 3.21 | .083 |
| Learning mode within 1 Mo by mwithin Session 7 | 1.25 | 1 | 1.25 | .05 | .817 |
| Learning mode within 12 Mo by mwithin Session 7 | 60.46 | 1 | 60.46 | 2.63 | .115 |
| Learning mode within 1 Mo by mwithin Session 8 | 48.05 | 1 | 48.05 | 3.13 | .087 |
| Learning mode within 12 Mo by mwithin Session 8 | 134.23 | 1 | 134.23 | 8.74 | .006** |
| Learning mode within 1 Mo by mwithin Session 9 | 14.45 | 1 | 14.45 | 1.56 | .221 |
| Learning mode within 12 Mo by mwithin Session 9 | 89.47 | 1 | 89.47 | 9.67 | .004** |
| Learning mode within 1 Mo by mwithin Session 10 | 12.8 | 1 | 12.8 | .38 | .541 |
| Learning mode within 12 Mo by mwithin Session 10 | 88.78 | 1 | 88.78 | 2.65 | .114 |
| Learning mode within 1 Mo by mwithin Session 11 | .20 | 1 | .20 | .03 | .859 |
| Learning mode within 12 Mo by mwithin Session 11 | 64.07 | 1 | 64.07 | 10.21 | .003** |
| Learning mode within 1 Mo by mwithin Session 12 | 1.80 | 1 | 1.80 | .17 | .687 |
| Learning mode within 12 Mo by mwithin Session 12 | 29.38 | 1 | 29.38 | 2.70 | .110 |

| Table S2 Simple-effect comparison on the number of days to learning success between different age and learning mode groups (Learning Stage) | | | | | |
| --- | --- | --- | --- | --- | --- |
|  | *SS* | *DF* | *MS* | *F* | *p* |
| Age within IL | 84.02 | 1 | 84.02 | 9.80 | .004^**^ |
| Age within PL | 7.04 | 1 | 7.04 | .82 | .372 |
| Learning mode within 1 Mo | 72.20 | 1 | 72.20 | 8.07 | .008^**^ |
| Learning mode within 12 Mo | 6.45 | 1 | 6.45 | .72 | .402 |

| Table S3 Simple-effect comparison on the total errors made between learning modes controlling for sessions (Learning Stage: Sub-stage One; 12 Mo groups) | | | | | |
| --- | --- | --- | --- | --- | --- |
|  | *SS* | *DF* | *MS* | *F* | *p* |
| Mwithin IL by Session 1-3 | 803.58 | 2 | 401.79 | 11.95 | .000^***^ |
| Mwithin PL by Session 1-3 | 4.67 | 2 | 2.33 | .07 | .933 |
| Learning mode by Mwithin Session 1 | 1106.30 | 1 | 1106.30 | 16.13 | .001^**^ |
| Learning mode by Mwithin Session 2 | 123.43 | 1 | 123.43 | 4.04 | .066 |
| Learning mode by Mwithin Session 3 | 27.14 | 1 | 27.14 | 1.32 | .271 |

| Table S4 Simple-effect comparison on the total errors made between learning modes controlling for sessions (Learning Stage; 1 Mo groups) | | | | | |
| --- | --- | --- | --- | --- | --- |
|  | *SS* | *DF* | *MS* | *F* | *p* |
| Mwithin IL by Session 1-3 | 560.07 | 2 | 280.03 | 22.79 | .000^***^ |
| Mwithin PL by Session 1-3 | 8.87 | 2 | 4.43 | .36 | .700 |
| Learning mode by Mwithin Session 1 | 649.80 | 1 | 649.80 | 32.82 | .000^***^ |
| Learning mode by Mwithin Session 2 | 120.05 | 1 | 120.05 | 7.33 | .014^*^ |
| Learning mode by Mwithin Session 3 | 26.45 | 1 | 26.45 | 11.87 | .003^**^ |
| Mwithin IL by Session 4-7 | 1.90 | 3 | .63 | .15 | .927 |
| Mwithin PL by Session 4-7 | 114.88 | 3 | 38.29 | 9.29 | .000^***^ |
| Learning mode by Mwithin Session 4 | 68.45 | 1 | 68.45 | 10.22 | .005^**^ |
| Learning mode by Mwithin Session 5 | 57.80 | 1 | 57.80 | 6.80 | .018^*^ |
| Learning mode by Mwithin Session 6 | 1.25 | 1 | 1.25 | 1.55 | .229 |
| Learning mode by Mwithin Session 7 | 1.25 | 1 | 1.25 | 1.27 | .274 |
| Mwithin IL by Session 8-12 | 1.60 | 4 | .40 | .23 | .919 |
| Mwithin PL by Session 8-12 | 64.72 | 4 | 16.18 | 9.39 | .000^***^ |
| Learning mode by Mwithin Session 8 | 48.05 | 1 | 48.05 | 16.35 | .001^**^ |
| Learning mode by Mwithin Session 9 | 14.45 | 1 | 14.45 | 4.19 | .056 |
| Learning mode by Mwithin Session 10 | 12.80 | 1 | 12.80 | 6.33 | .022^*^ |
| Learning mode by Mwithin Session 11 | .20 | 1 | .20 | .90 | .355 |
| Learning mode by Mwithin Session 12 | 1.80 | 1 | 1.80 | 3.86 | .065 |

| Table S5 Results of non-repeated measures analysis of variance of the number of total errors made by different phases of the experiment, learning modes and ages | | | | | | | |
| --- | --- | --- | --- | --- | --- | --- | --- |
|  | | *SS* | *DF* | *MS* | *F (DFn, DFd)* | *p* | η2 |
| Phase of the experiment | 733.624 | | 2 | 366.812 | *F* (2, 62) = 24.291 | .000*** | 0.439 |
| Learning mode | | 18.398 | 1 | 18.398 | *F* (1, 31) = 0.884 | .354 | 0.028 |
| Age | 939.761 | | 1 | 939.761 | *F* (1, 31) = 45.139 | .000*** | 0.593 |
| Phase × Age | | 42.854 | 2 | 21.427 | *F* (2, 62) = 1.419 | .250 | 0.044 |
| Phase × Learning mode | | 68.271 | 2 | 34.135 | *F* (2, 62) = 2.261 | .113 | 0.068 |
| Learning mode×Age | | 12.96 | 1 | 12.96 | *F* (1, 31) = 0.622 | .436 | 0.020 |
| Phase×Learning mode×Age | | 110.25 | 2 | 55.125 | *F* (2, 62) = 3.651 | .032* | 0.105 |

| Table S6 *Post-hoc* multiple comparisons among different phases of the experiment | | | | | |
| --- | --- | --- | --- | --- | --- |
|  |  | *MD* | *SE* | *p* | 95%CI |
| Phase of the experiment | Session 8-12 of Learning vs Retest | -0.709 | 0.421 | .102 | [-1.57, 0.15] |
|  | Retest vs Gestalt transfer learning | -5.285 | 1.121 | .000*** | [-7.57, -3.00] |
|  | Session 8-12 of Learning vs Gestalt transfer learning | -5.994 | 1.102 | .000*** | [-8.24, -3.75] |

| Table S7 *Post-hoc* multiple comparisons among different age groups | | | | | |
| --- | --- | --- | --- | --- | --- |
|  |  | *MD* | *SE* | *p* | 95%CI |
| Age | 1 Mo vs 12 Mo | -6.053 | 0.901 | .000^***^ | [-7.89, -4.22] |

| Table S8 Simple-effect comparisons on total errors made between learning modes and ages within/among different test stages | | | | | |
| --- | --- | --- | --- | --- | --- |
|  | *SS* | *DF* | *MS* | *F* | *p* |
| Learning mode within 1 Mo by Mwithin Session 8-12 | 10.37 | 1 | 10.37 | 1.48 | .233 |
| Learning mode within 12 Mo by Mwithin Session 8-12 | 77.06 | 1 | 77.06 | 11.01 | .002^**^ |
| Learning mode within 1 Mo by Mwithin Retest stage | .45 | 1 | .45 | .05 | .827 |
| Learning mode within 12 Mo by Mwithin Retest stage | 5.36 | 1 | 5.36 | .58 | .451 |
| Learning mode within 1 Mo by Mwithin Gestalt stage | 43.02 | 1 | 43.02 | 1.24 | .275 |
| Learning mode within 12 Mo by Mwithin Gestalt stage | 69.79 | 1 | 69.79 | 2.01 | .167 |
| Age within IL by Mwithin Session 8-12 | 36.61 | 1 | 36.61 | 5.23 | .029^*^ |
| Age within PL by Mwithin Session 8-12 | 120.63 | 1 | 120.63 | 17.23 | .000^***^ |
| Age within IL by Mwithin Retest stage | 235.86 | 1 | 235.86 | 25.59 | .000^***^ |
| Age within PL by Mwithin Retest stage | 168.20 | 1 | 168.20 | 18.25 | .000^***^ |
| Age within IL by Mwithin Gestalt stage | 487.16 | 1 | 487.16 | 14.00 | .001^**^ |
| Age within PL by Mwithin Gestalt stage | 62.14 | 1 | 62.14 | 1.79 | .191 |
| Mwithin IL within 1 Mo by test stages | 96.27 | 2 | 48.13 | 3.19 | .048^*^ |
| Mwithin IL within 12 Mo by test stages | 559.73 | 2 | 279.87 | 18.53 | .000^***^ |
| Mwithin PL within 1 Mo by test stages | 235.69 | 2 | 117.84 | 7.80 | .001^**^ |
| Mwithin PL within 12 Mo by test stages | 59.77 | 2 | 29.88 | 1.98 | .147 |
